# Harnessing spin and orbital angular momentum light for optimal algae growth

**DOI:** 10.1038/s41598-024-56203-1

**Published:** 2024-04-12

**Authors:** Yancong He, Ziling Huang, Qiongfang Zeng, Huihui Huang

**Affiliations:** 1 Key Laboratory for Micro/Nano Optoelectronic Devices of Ministry of Education and Hunan Provincial Key Laboratory of Low-Dimensional Structural Physics and Devices, School of Physics and Electronics, Hunan University, Changsha, 410082 China; 2 School of Public Administration and Human Geography, Hunan University of Technology and Business, Changsha, 410205 China

**Keywords:** Biophysics, Biological physics

## Abstract

The present study investigated the difference in transmittance of light carrying opposite spin angular momentum (SAM) and orbital angular momentum (OAM) through chlorella algal fluid with varying concentrations and thicknesses. Our results indicate that, under specific conditions, right-handed light sources exhibit higher transmittance in the algal fluid compared to left-handed light sources. Furthermore, we observed that light with OAM also demonstrated higher transmittance than other types of light sources, leading to faster cell density growth of Chlorella. Interestingly, we also discovered that light with OAM stimulates Chlorella to synthesize more proteins. These findings provide different insights for selecting appropriate light sources for large-scale algae cultivation, and may facilitate the realization of carbon peaking and carbon neutrality in the future.

## Introduction

Optical angular momentum, which encompasses both spin angular momentum (SAM) and orbital angular momentum (OAM), is a measurable attribute of an individual photon^1^. Among them, the SAM is related to the polarization state of light, while the OAM is included in the Laguerre-Gaussian(LG) beam containing spiral phase wavefront pioneered by Allen and others in 1992^2^.

**Figure 1:**
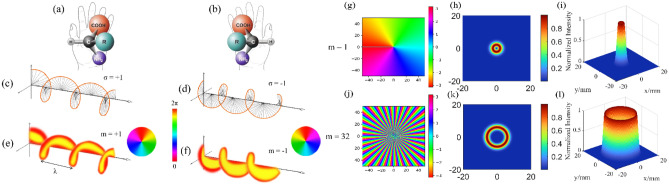
Schematic diagram of chiral amino acid molecules and chiral light sources. (**a**,**b**) Schematic diagram of a pair of chiral amino acid molecules. (**c**,**d**) Schematic diagram of the propagation of the electric field vector and magnetic field vector of two CPL with opposite rotations in space. (**e**,**f**) Schematic diagram of helical phase wavefront propagation of a LG beam with topological charge $m=\pm 1$ *in* one period. (**g**–**i**) The spiral phase diagram and 2D and 3D light field intensity distribution diagram of a ring vortex with a topological charge number of *m* = *1*. (**j**–**l**) Spiral phase diagram and 2D and 3D light field intensity distribution diagram of a ring vortex with a topological charge number of *m* = *32*.

Chiral isomers refer to pairs of molecules with identical composition and atomic arrangement, but possessing a mirror image relationship akin to left and right hands. These molecules are unable to overlap in three-dimensional space and represent a ubiquitous phenomenon in nature^3^. Molecules featuring chiral isomers are known as chiral molecules. Figure 1a,b illustrates the chiral amino acid molecules which, despite sharing the same molecular formula, cannot completely superimpose in space.

As an electromagnetic wave, light possesses the ability to exhibit chirality through its angular momentum. This phenomenon can be observed in Fig. 1c,d, where circularly polarized light(CPL) with opposing SAM will cause their electric and magnetic field vectors to rotate either clockwise (left-hand) or counterclockwise (right-hand).

CPL, owing to its SAM, has the ability to preferentially interact with one of the enantiomers of chiral substances resonant with electromagnetic waves^4^. The absorption coefficients of the same substance might exhibit variations when interacting with Left-handed Chiral Photons(L-CP) and Right-handed Chiral Photons(R-CP)^5^. The disparity between these coefficients is referred to as Circular Dichroism (CD), which has found extensive use in identifying chiral molecules in both chemical and biological settings. Kuhn's g-factor^6^ is a well-known parameter associated with CD, which can be expressed as follows^7^

1 $${\text{g}}=\frac{\langle {\Gamma }^{{\text{L}}}\rangle -\langle {\Gamma }^{{\text{R}}}\rangle }{\frac{1}{2}(\langle {\Gamma }^{{\text{L}}}\rangle +\langle {\Gamma }^{{\text{R}}}\rangle )},$$

where $\langle {\Gamma }^{{\text{L}}}\rangle$ represents the absorption rate of L-CP, while $\langle {\Gamma }^{{\text{R}}}\rangle$ represents the absorption rate of R-CP. The coefficient refers to the relative difference between the absorption coefficient of L-CP and R-CP of the substance in the Absorption band, which can be used to reflect the intensity of CD signal.

Similar to the SAM, an optical vortex carrying orbital angular momentum (OAM) also exhibits chirality^8^, which is encoded in its helical phase front. In Fig. 1e, the topological charge of the LG beam is $m=+1$, so its spiral phase wave front propagation direction rotates clockwise, which is similar to Left-handed Circularly Polarized Light(LCPL) with SAM $\sigma =+1$, both of which are left-handed. We can refer to this vortex light with a left-handed spiral phase wavefront as a left-handed vortex light (LVL).

On the contrary, in Fig. 1f, the LG beam with topological charge $m=-1$ is similar to the Right-handed Circularly Polarized Light(RCPL) with SAM $\upsigma =-1$, and they are all right-handed. Similar to LVL, we can refer to this vortex light with a right-handed spiral phase wavefront as a right-handed vortex light (RVL). Similar to Kuhn's g-factor for CD measurements, J. Ni and colleagues defined the vortical differential.

Scattering (VDS) using the dissymmetry factor as follows^9^:

2 $${\text{VDS}}=\frac{{{\text{I}}}_{+\left|{\text{m}}\right|}-{{\text{I}}}_{-\left|{\text{m}}\right|}}{\frac{1}{2}({{\text{I}}}_{+\left|{\text{m}}\right|}+{{\text{I}}}_{-\left|{\text{m}}\right|})},$$

where ${I}_{+\left|m\right|}$ and ${I}_{-\left|m\right|}$ are the scattering intensity under the illumination of optical vortices with topological charge +*|m|* and −*|m|*, respectively. Their research also proves that LG beams carrying OAM can also detect chiral substances similar to CPL.

For vortex beams with varying topological charges, an increase in the number of topological charges, or higher order, results in a larger dark central core within the optical field, along with an overall increase in the optical field area^10^. This phenomenon is effectively demonstrated in Fig. 1g–l through a comparative analysis of vortex light examples with topological charge numbers of *m* =  ± *1* and *m* =  ± *32*.

Optical vortices have been a hot research field in recent years. In the past two years, this field has achieved a series of highly valuable achievements in combination with liquid crystal orientation^11^, optical edge detection^12^, and other fields. As research has progressed, the previous challenges of developing electrically tailored working bands^13^ and tunable band-pass optical vortex processors enabled by wash-out-refill chiral superstructures^14^, as well as vortex laser arrays equipped with topological charge control and self-healing of defects^15^, have been gradually overcome by researchers.

Previous studies have shown that under certain conditions, the transmittance of LG beams in certain biological tissues and scattering media is higher than that of ordinary lasers^16–18^. Due to the scattering and biological tissue characteristics of Chlorella fluid, we also tested its transmittance using several different types of light sources. Distinguishing ourselves from prior studies, our investigation focused not only on evaluating the transmittance discrepancies between OAM and SAM light sources in Chlorella fluid but also on assessing the variations in transmittance for light sources with different chiralities within Chlorella fluid.

Among numerous biomass, microalgae have the advantages of high lipid content, high biomass yield per unit area, short growth cycle and easy cultivation. These characteristics make them an excellent source material for producing biomass fuels^19^, as well as for applications such as carbon dioxide fixation^19–21^ and wastewater treatment^22^. Among the various species of microalgae, Chlorella stands out as a particularly important research subject^19–22^. Previous studies on the growth of Chlorella under different lighting conditions have mainly focused on the wavelength^23^ or spectrum^24^ of the light source, as well as the intensity^24,25^ and light–dark cycle^26^. However, very little attention has been paid to the polarization or vortex state of the light source, specifically whether it possesses SAM or OAM.

Hence, subsequent to conducting the transmittance detection experiment, we proceeded to devise an algae culture experiment aiming to examine the potential correlation between enhanced transmittance in Chlorella algae fluid and the promotion of Chlorella growth. Additionally, our objective was to investigate the influence of light sources employing Orbital Angular Momentum (OAM) on both Chlorella growth and organic synthesis.

## Experiment

### Algae preparation

We procured 1L of Chlorella fluid (strain FACHB-10) from the freshwater algae seed bank of the Chinese Academy of Sciences and partitioned it into two fractions. One fraction was cultivated to attain a specific density of algae cells and subsequently diluted to concentrations of 1.7 M, 3 M, 8 M, 17 M, and 52 M cells/ml for transmission testing experiment. The remaining fraction will undergo cultivation until the logarithmic growth phase is reached. Subsequently, it will be divided into five equal portions with equivalent volumes and approximately uniform algae cell densities to be utilized for algal culture experiment.

### Transmission testing experiment

We conducted transmittance tests on four different types of light sources in Chlorella algae fluid with different cell densities and thicknesses.

**Figure 2:**
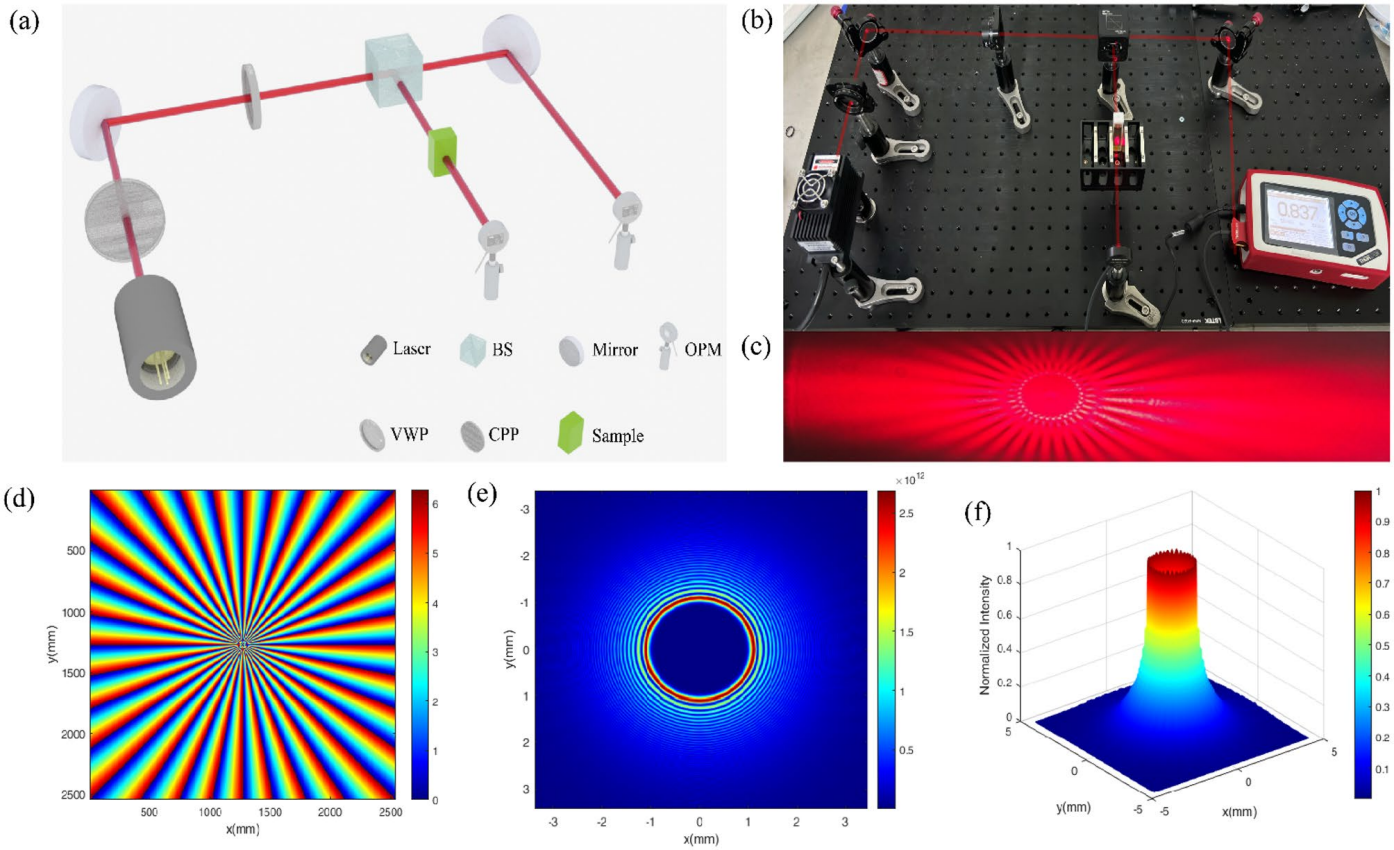
Schematic diagram of optical path, physical image, and vortex light effect diagram with topological charge number m =  ± 32 for transmittance testing experiment. (**a**) A schematic illustration depicting the optical pathway utilized for transmittance testing experiments, employing vortex light generation as an exemplar. (**b**) Physical image of the optical path corresponding to (**a**). (**c**) Effect image of vortex light generated in the optical path (m =  + 32). (**d**–**f**) The measured Spiral phase diagram and 2D and 3D light field intensity distribution diagram of the vortex beam with topological charge m =  + 32 generated in (**c**), respectively.

The tested light sources included LCPL, RCPL, and two kinds of vortex beams with a topological charge of ± 32, all of which had a wavelength of 670 nm. It should be noted that the vortex light used in our study was produced by a vortex wave plate that has a topological charge of *m* =  ± *32*, using circularly polarized light as the input. This results in the light possessing both SAM and OAM. And we selected a vortex laser with a higher absolute topological charge (*m* =  ± *32*) based on our literature review which indicated that greater topological charges are associated with enhanced transmittance of vortex lasers in biological tissues^16^. To optimize experimental outcomes and minimize equipment expenses, we ultimately chose two topological charges, both with *m* =  ± *32*. Furthurmore, experiments were carried out on five different algal cell densities ranging from 1.7 M to 5.2 M cells/ml, as well as three different algal fluid thicknesses of 10 mm, 20 mm, and 30 mm. The experimental optical path diagram for transmittance testing is shown in Fig. 2.

Figure 2a depicts the experimental light path diagram using the example of the light path generating the vortex beam. Among these, lasers, circular polarization plate (CPP), and vortex wave plates (VWP) were utilized to generate four distinct types of laser light sources for testing purposes. A non-polarized 50:50 beam splitting cubic beam splitter (BS) was employed in the experimental setup. The purpose of using the beam splitter is to divide the generated light source into two equal intensity parts without altering its polarization state. This facilitates the use of optical power meter (OPM) for transmittance testing of algal fluid samples. Subfigure (b) portrays the physical representation of the aforementioned optical pathway illustrated in Fig. 2a. Subfigure (c) displays the effect of the vortex light generated within the optical pathway.

Additionally, to confirm the topological charge of the generated vortex light as ± 32, interference was utilized to produce its spiral phase diagram, as depicted in Fig. 2d, where *m* =  + *32* was taken as an example. Analysis of the interference pattern revealed 32 cycles of phase rotation within a 360° range, thus confirming the topological charge of the generated vortex light as 32. Furthermore, Fig. 2e and f present the two-dimensional and three-dimensional intensity distribution of the light field, respectively, displaying prominent phase singularities in the central region.

### Algae culture experiment

The Chlorella vulgaris samples, which had entered the logarithmic growth phase, were divided into five equal portions and placed in conical flasks of identical size for cultivation. Subsequently, we subjected them to different light sources with wavelengths around 670 nm, namely LED, LCPL, RCPL, and *m* =  ± *32* vortex light sources. The experimental arrangement is depicted in Fig. 4a. The temperature was maintained at approximately 25 ℃, with a light–dark cycle of 12 h on and 12 h off, accompanied by a light intensity of approximately 2000 lx. Additionally, we conducted cell density and chlorophyll concentration measurements for each group every two days.

### Algae cell density measurement

We employed the cell count technique to determine the density of chlorella cells. The procedure consists of several steps. Firstly, samples were acquired by obtaining five aliquots of Chlorella solution, which were subsequently diluted by a factor of 100–200. Secondly, 0.1 ml of the diluted algae solution was quantitatively extracted and introduced onto a glass slide containing a counting box. The slide was covered and positioned on the optical microscope stage. The third step involved adjusting the microscope magnification to 100 × and enumerating the Chlorella cells within the counting box. Finally, a conversion process was conducted. The Chlorella count within the counting box was multiplied by 10 and then further multiplied by the dilution factor to determine the density of algal cells (cells/ml).

### Chlorophyll concentration measurement

The chlorophyll concentration of Chlorella algae fluid was measured using spectrophotometry. The experimental procedures included the following steps:

1. Sampling: A total of 8 ml of algae fluid was extracted from each of the five sample groups and transferred to individual centrifuge tubes.
2. Centrifugation: The centrifuge tubes were placed in a centrifuge and subjected to centrifugation at a speed of 10,000r/min for 10 min.
3. Filtration: The resulting algae fluid from the five groups were individually filtered using a 0.45 μm filter, and the filter residues were collected on filter paper.
4. Ethanol treatment: The filter residues from each group were transferred to separate 10 ml test tubes. Subsequently, 8 ml of anhydrous ethanol solution was added to each test tube. The test tubes were then placed in a constant temperature water bath at 60 ℃ for 6 h.
5. Centrifugation: The test tubes containing the ethanol-treated solutions were centrifuged at a speed of 4000r/min for 10 min.
6. Filtration: The solutions from the five groups, which had undergone centrifugation, were filtered, and the filtrates were collected in five colorimetric dishes.
7. Blank control: Anhydrous ethanol solution was added to the sixth colorimetric dish as a blank control group.
8. Absorbance measurement: The absorbance of the six cuvettes, including the five samples and the blank control, was measured at wavelengths of 649 nm, 665 nm, and 750 nm. The values were recorded.
9. Calculation: The concentration of chlorophyll a, chlorophyll b, and total chlorophyll was calculated using the following formula:
  $$Ch{l}_{a}=13.7\left({A}_{665}-{A}_{750}\right)-5.76\left({A}_{649}-{A}_{750}\right),$$
  $$Ch{l}_{b}=25.8\left({A}_{649}-{A}_{750}\right)-7.6\left({A}_{665}-{A}_{750}\right),$$
  3 $$Chl= Ch{l}_{a}+ Ch{l}_{b},$$

where ${A}_{649}$, ${A}_{665}$, and ${A}_{750}$ refer to the absorbance of the sample at 649 nm, 665 nm, and 750 nm, respectively.

### Measurement of the proportion of biological macromolecule content

After the completion of cultivation, we conducted mid infrared spectroscopy tests on each group of algae mud, The results of these tests are depicted in Fig. 4b. Based on spectral analysis, it is evident that the absorption peaks within the range of 2800–3000 cm^–1^ correspond to the oil content in the biomass, while the absorption peaks between 1500 and 1700 cm^–1^ originate from protein content in the biomass. Furthermore, the absorption peaks within the range of 1000–1200 cm^–1^ correspond to polysaccharides present in the biomass. By classifying the substances and dividing the spectral range, we were able to obtain the component contents of the three biological macromolecules through the calculation of characteristic peak areas. These findings are illustrated in Fig. 4c.

## Results

**Figure 3:**
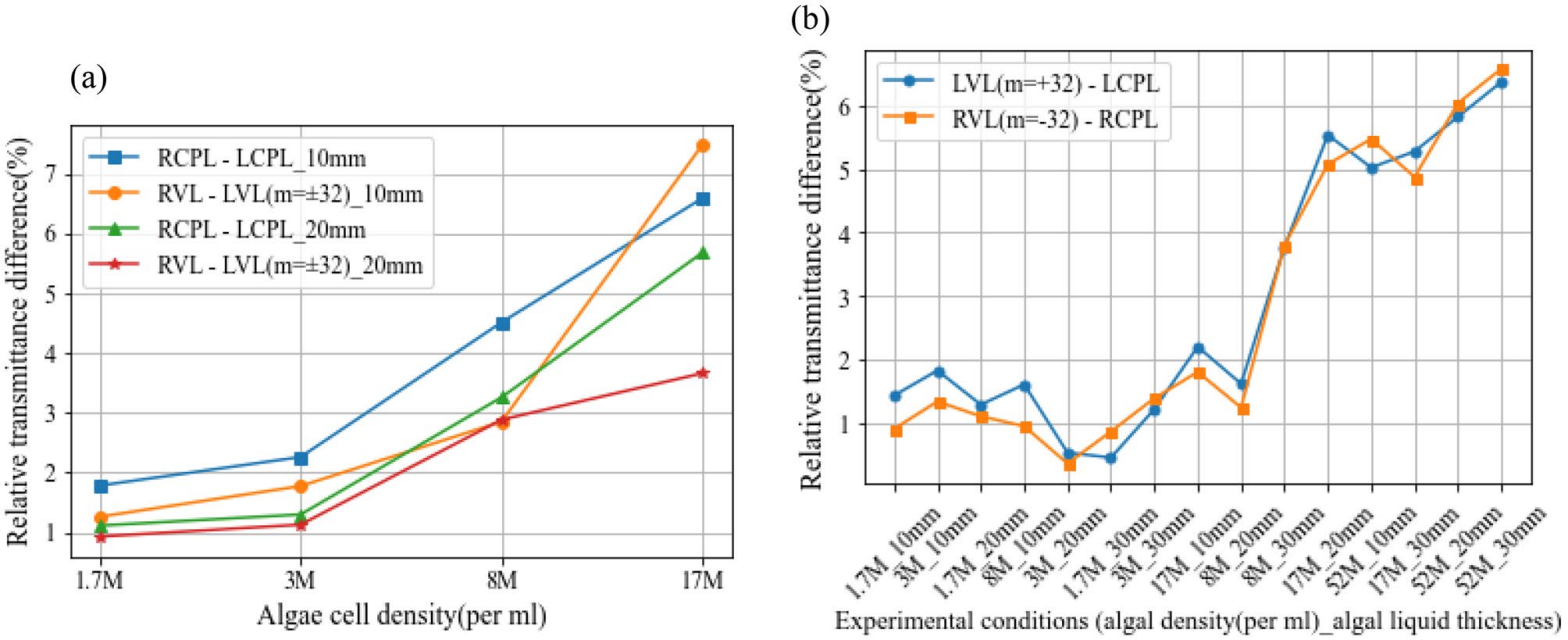
Results of the transmittance experiment. (**a**) The relative difference in transmittance between RCPL, LCPL, vortex beams with *m* =  *− 32* and *m* =  + *32* is shown for Chlorella algal fluid with varying concentrations and thicknesses. The legend describes the relative transmittance difference between two light sources of opposite rotation in a specific thickness of Chlorella algal fluid. The term before the underline denotes the transmittance difference, while the term after the underline indicates the algal solution thickness. For instance, ‘RCPL–LCPL_10mm’ represents the relative transmittance difference between RCPL and LCPL light sources in a 10 mm thick Chlorella algal fluid. See Data File 1 for underlying values. (**b**) The relative difference in transmittance between votex beams with the same rotation direction and CPL under different experimental conditions. In the legend, ‘LVL (*m* =  + *32*) – LCPL’ refers to the difference in transmittance between LVL with *m* =  + *32* and LCPL under various test conditions in Chlorella algae solution. ‘RVL (*m* =  *− 32*) – RCPL’ has a similar meaning. See Data File 2 for underlying values.

The experimental results of the transmission testing experiment are shown in Fig. 3a,b.

Based on the findings of Fig. 3a,b, it was observed that, when subjected to the experimental conditions, right-handed light and light carrying OAM exhibited higher transmittance in Chlorella algae fluid compared to left-handed light and light carrying only SAM. Based on our findings, it is hypothesized that light with a wavelength close to 670 nm, exhibiting right-handed chirality and carrying OAM, may be more favorable for the growth of Chlorella. Consequently, a subsequent experiment was promptly conducted to explore the potential implications of these findings on the selection of light sources in Chlorella cultivation. The details of this investigation are discussed in the subsequent section of this article.

Based on the data presented in Tables 1 and 2, it is evident that under the cultivation of four light sources with SAM or OAM, the density of algal cells and the concentration of chlorophyll of Chlorella grow faster than that of LED light source. Among them, the growth rate of Chlorella density was found to be 63.2% higher in the vortex light cultivation with *m* = *− 32* compared to the LED cultivation. Furthermore, the density of algal cells and chlorophyll concentration are observed to grow faster under the two groups of cultivation light sources with OAM than those with only SAM. Meanwhile, the growth rate of Chlorella under right-handed light culture is higher than that under left-handed light culture. We believe that the above experimental results are likely caused by the higher transmittance of right-handed light and vortex light in Chlorella algae fluid.

**Table 1:** Comparison of partial algal cell density (cells/mL) data and total growth rate for each group.

Light sources	Day 1	Day 15	Growth rate
LED	2.87M	8.86M	302.4%
LCPL	2.82M	9.21M	327.2%
RCPL	2.19M	9.35M	426.9%
m = + 32 vortex light	2.25M	9.50M	422.2%
m = -32 vortex light	2.01M	9.92M	493.5%

**Table 2:** Comparison of partial chlorophyll concentration (mg/L) data and total growth rate for each group.

Light sources	Day 1	Day 15	Growth rate
LED	0.2518	0.6953	276.1%
LCPL	0.2814	0.8182	288.0%
RCPL	0.2814	0.7537	265.3%
m = + 32 vortex light	0.2257	0.7659	339.3%
m = − 32 vortex light	0.2640	0.8626	326.7%

**Figure 4:**
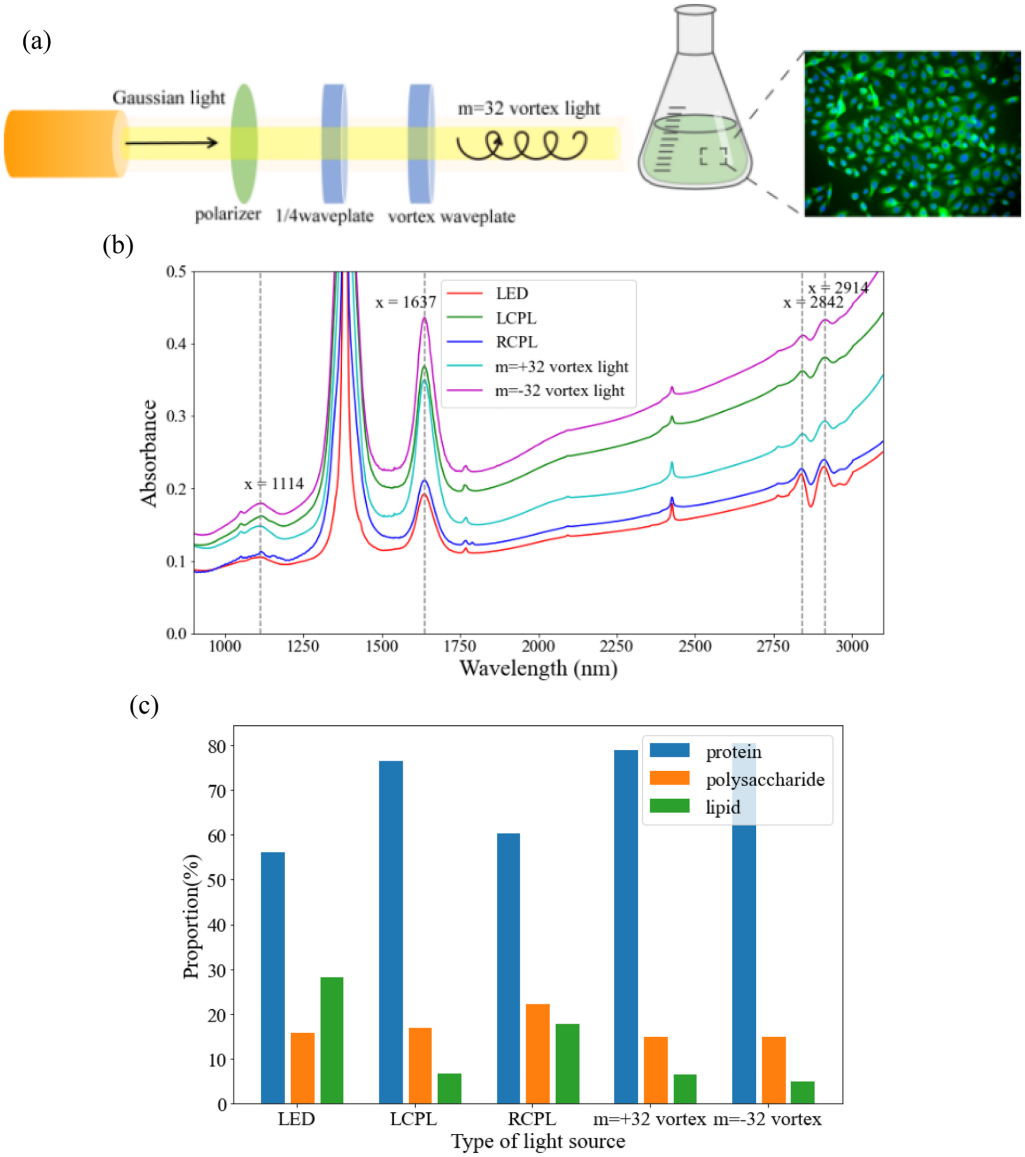
Culture experiment of Chlorella using different types of light sources. (**a**) Schematic diagram of the experimental setup. (**b**) The mid infrared spectra of the algae mud from the five groups tested after the experiment. See Data File 3 for underlying values. (**c**) Histogram of the proportion of biomacromolecule content in each group obtained through mid infrared spectroscopy analysis. See Data File 4 for underlying values.

The experimental arrangement is depicted in Fig. 4a. Figure 4b illustrates the mid infrared spectra of algal mud obtained after the experiment, corresponding to each culture group. Additionally, Fig. 4c displays a histogram showcasing the distribution of biomacromolecule content proportions, which were derived from Fig. 4b analysis.

The results illustrated in Fig. 4c demonstrate that Chlorella exposed to light with OAM has a higher protein synthesis rate, while LED light induces a higher lipid synthesis rate. This finding is consistent with our previous research, where we observed that vortex light promotes hair growth in mice^10^, which is closely associated with protein synthesis.

## Discussion

In the preceding section, a critical finding was derived from the experimental outcomes depicted in Fig. 2. Specifically, our investigation revealed that, right-handed light and light carrying OAM exhibited higher transmittance in Chlorella algae fluid compared to left-handed light and light carrying only SAM. Moreover, we also found in the algae culture experiment that right-handed light sources and light sources carrying OAM can promote faster growth of Chlorella algal fluid. These cornerstone discoveries underpin the entirety of our research endeavor, thus necessitating an in-depth exploration of the underlying factors contributing to this phenomenon in the following section.

Firstly, we will discuss the reasons why right-handed light has a higher transmittance in Chlorella algae fluid than left-handed light and makes the Chlorella grow faster under the cultivation with a right-handed light source. Prior to conducting the algal culture experiment, we performed visible light circular dichroism (CD) spectroscopy tests on two distinct densities of Chlorella algae fluid.

**Figure 5:**
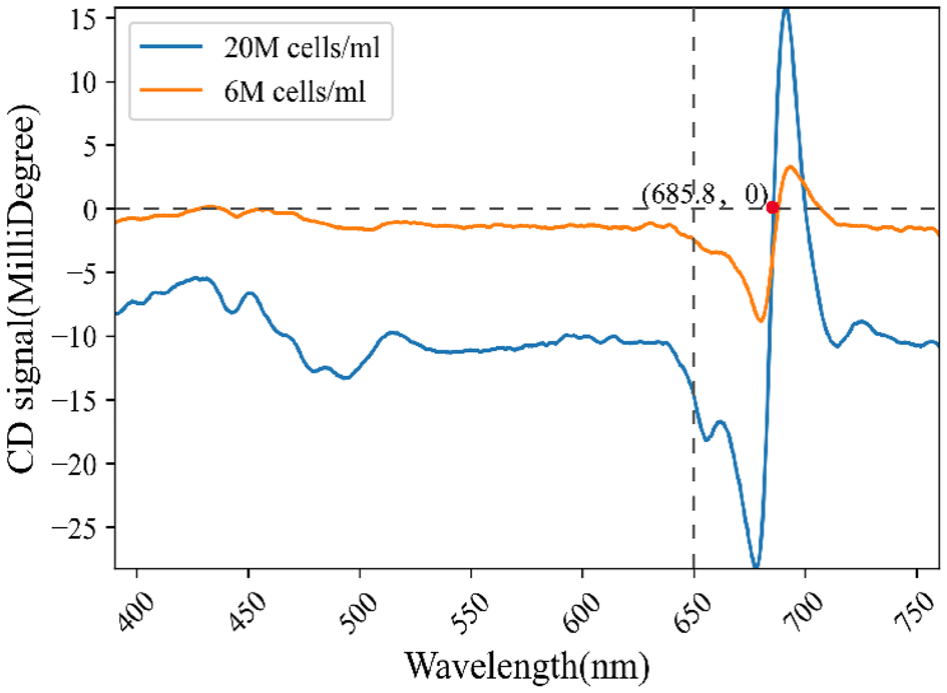
Circular dichroism spectra of Chlorella fluid with algal cell densities of 6M cells/mL and 20M cells/mL, respectively.

It can be seen from Fig. 5 that the algal fluid of Chlorella has an obvious CD signal in the wavelength range of 650–700 nm. And the CD signal is stronger when the density of algal cells is larger, which can also account for the observed increase in the disparity of transmittance between the left and right chiral light sources, as depicted in Fig. 4a, with the elevation of Chlorella fluid concentration. And we can know that the CD signal is negative in the wavelength range of 650–685 nm.

Hence, it is deemed that the Chlorella fluid synthesized in our experiment displays a significantly enhanced degree of dextral chirality when subjected to 670 nm incident light, leading to heightened interactions with right-polarized light sources^9^. We posit that this is a plausible explanation for the observed accelerated growth rate of Chlorella cultures exposed to right-handed light.

Secondly, it is imperative to examine the reasons why the transmittance of light sources bearing OAM through Chlorella fluid surpasses that of light sources lacking OAM. As previously stated, we selected a topological charge of vortex light with m =  ± 32 based on prior research demonstrating that as the topological charge of vortex light increases, its transmittance in scattering media such as chicken breast tissue^16^ and scattering medium^17^ also increases. To elucidate the dissimilarities in transmittance between vortex beams and Gaussian lasers in microalgae solution, it is crucial to establish a scattering model of vortex beams in underwater environments and investigate the scattering characteristics of suspended spherical algal particles when exposed to vortex beams.

Wang Mingjun et al. have developed a scattering model for underwater suspended spherical algae particles based on the generalized Mie theory and Laguerre Gaussian vortex beams^27^. Their study employed numerical simulation to demonstrate that the differential scattering cross-section between suspended spherical algal particles and Laguerre Gaussian vortex beams decreases as the topological charge of the beam increases. Their theory precisely accounts for our experimental findings, which indicate that higher topological charges of the vortex beam lead to a larger central hollow region in the light field, resulting in a smaller differential scattering cross-section with the suspended spherical algal particle group and a consequent increase in transmittance in the microalgae fluid.

Furthermore, the precise mechanism underlying the ability of vortex light to enhance the biosynthesis of chlorophyll and proteins in microalgae remains unclear, and it is our hopeful expectation that this matter can be resolved in future research.

## Conclusion

In summary, our research findings demonstrate a higher transmittance of right-handed light and light carrying OAM in Chlorella algae fluid compared to left-handed light and light carrying only SAM. Additionally, our results suggest that right-handed light and light with OAM are more suitable for cultivating Chlorella. And the aforementioned outcomes were analyzed and discussed to elucidate their underlying rationales. Moreover, we observed that light with OAM promotes the protein synthesis of Chlorella. However, the precise mechanisms underlying the favorable effects of vortex light on the enhancement of chlorophyll and protein synthesis remain unknown. This knowledge gap will be a significant focus of our future research endeavors. Ultimately, our discovery is expected to offer valuable insights into the selection of light sources for large-scale algal cultivation and contribute to achieving carbon peaking and carbon neutrality efforts as soon as possible.

### Supplementary Information

Supplementary Information 1.

Supplementary Information 2.

Supplementary Information 3.

Supplementary Information 4.

Supplementary Information 5.

Supplementary Information 6.

## Data Availability

All data generated or analysed during this study are included in this published article [and its supplementary information files].
