# Supplementary material for: Harnessing spin and orbital angular momentum light for optimal algae growth
Source: Sci Rep. 2024 Apr 12;14:8564. doi: 10.1038/s41598-024-56203-1 (PMC11014974; doi:10.1038/s41598-024-56203-1)
Supplement: Supplementary file 6 — Supplementary Information 6. [file 41598_2024_56203_MOESM6_ESM.docx]

**Supplementary Information for**

**Harnessing Spin and Orbital Angular Momentum Light for Optimal Algae Growth**

**Yancong He^1^, Ziling Huang^1^, Qiongfang Zeng^2🖂^ ＆ Huihui Huang ^1🖂^**

^1^Key Laboratory for Micro/Nano Optoelectronic Devices of Ministry of Education and Hunan Provincial Key Laboratory of Low-Dimensional Structural Physics and Devices, School of Physics and Electronics, Hunan University, Changsha 410082, China. ^🖂^email: [huangh@hnu.edu.cn](mailto:huangh@hnu.edu.cn)

^2^School of Public Administration and Human Geography, Hunan University of Technology and Business, Changsha 410205, China. *email: [2681@hutb.edu.cn](mailto:2681@hutb.edu.cn)

**Supplementary Information Text**

**Note S1. A brief introduction to Kuhn's g-factor**

Kuhn refers to this factor as a anistropy-factor. It is the factor by which the contribution to ordinary refraction has to be multiplied in order to get the contribution to the optical activity^1^. Its initial form was

$g=\frac{{(\varepsilon_{\mathrm{LCP}}-\varepsilon_{RCP)}}_{\xi}}{{(\varepsilon)}_{\xi}}$ (1)

Where, ${(\varepsilon_{\mathrm{LCP}}-\varepsilon_{RCP)}}_{\xi}$ is the difference between the absorptivity of the material in the Absorption band and the left-right circularly polarized light, and ${(\varepsilon)}_{\xi}$ is the total absorptivity. This coefficient represents the relative difference of the absorption coefficients for Left-handed Circularly Polarized Light(LCPL) and Right-handed Circularly Polarized Light(RCPL) inside the absorption band (Circular Dichroism(CD)).

Since the absorption of LCPL and RCPL by the substance in the absorption band can actually be seen as the absorption of Left-handed Chiral Photons(L-CP) and Right-handed Chiral Photons(R-CP), the g factor can also be written as follows^2^

$g=\frac{\left\langle\Gamma^{L} \right\rangle-\left\langle\Gamma^{R} \right\rangle}{\frac{1}{2}(\left\langle\Gamma^{L} \right\rangle+\left\langle\Gamma^{R} \right\rangle)}$ (2)

where $\left\langle\Gamma^{L} \right\rangle$ represents the absorption rate of L-CP, while $\left\langle\Gamma^{R} \right\rangle$ represents the absorption rate of R-CP. This is Formula 1 in the main text.

From equation S1 and $\varepsilon=-lgT$:

$g=\frac{{(\varepsilon_{\mathrm{LCP}}-\varepsilon_{\mathrm{RCP}})}_{\xi}}{{(\varepsilon)}_{\xi}} =\frac{{(-lgT_{LCP}-\left( -lgT_{RCP} \right))}_{\xi}}{{(\varepsilon)}_{\xi}}=\frac{{(lgT_{RCP}-lgT_{LCP})}_{\xi}}{{(\varepsilon)}_{\xi}}$ (3)

Among them, T represents transmittance. Based on equation S4, it is apparent that a positive g-factor in the CD spectrum corresponds to a higher LCPL absorption rate and a higher RCPL transmittance for the sample. Conversely, a negative g-factor in the CD spectrum indicates a greater RCPL absorption and a greater LCPL transmittance for the sample at that particular wavelength.

**Note S2. Details of the transmittance testing experiment in the main text**

The tested light sources included LCPL, RCPL, and two kinds of vortex beams with a topological charge of ±32, all of which had a wavelength of 670nm. Notably, our vortex beam was generated based on circular polarization, and experiments were carried out on five different algal cell densities ranging from 1.7M to 5.2M cells/ml, as well as three different algal fluid thicknesses of 10mm, 20mm, and 30mm. The laser was preheated for a duration of 30 minutes. Subsequently, the prepared algal fluid was positioned between non-polarized 50:50 beam splitting cubic Beam Splitter (BS) and Optical Power Meter(OPM), with a distance of 30cm separating the two components. Refer to Fig. 2 in the main text for the optical path diagram.

The original data of the experiment are shown in Table S1, S2, and S3.It is important to mention that the initial data has been manipulated, wherein we designate the LCPL transmittance in chlorella liquid as 100%, while the transmittance readings of other light sources indicate the comparative transmittance.

**Table S1.** The relative transmittance of each light source in algal fluid with different densities of algal cells (thickness of algal fluid is 10mm).

| Algal cell density(cells/mL) | 1.70E+06 | 3.00E+06 | 8.00E+06 | 1.70E+07 |
| --- | --- | --- | --- | --- |
| LCPl | 100.00% | 100.00% | 100.00% | 100.00% |
| RCPL | 101.79% | 102.26% | 104.51% | 106.59% |
| m=+32 vortex light | 101.44% | 101.84% | 101.62% | 102.21% |
| m=-32 vortex light | 102.71% | 103.61% | 105.48% | 108.41% |
| RCPL-LCPL | 1.785% | 2.262% | 4.514% | 6.591% |
| RVL-LVL(m=±32) | 1.263% | 1.776% | 3.858% | 6.197% |

**Table S2.** The relative transmittance of each light source in algal fluid with different densities of algal cells (thickness of algal fluid is 20mm).

| Algal cell density(cells/mL) | 1.70E+06 | 3.00E+06 | 8.00E+06 | 1.70E+07 |
| --- | --- | --- | --- | --- |
| LCPl | 100.00% | 100.00% | 100.00% | 100.00% |
| RCPL | 101.12% | 101.30% | 103.26% | 105.68% |
| m=+32 vortex light | 101.30% | 100.55% | 101.63% | 105.53% |
| m=-32 vortex light | 102.23% | 101.68% | 104.52% | 110.75% |
| RCPL-LCPL | 1.115% | 1.301% | 3.260% | 5.681% |
| RVL-LVL(m=±32) | 0.932% | 1.131% | 2.886% | 5.221% |

**Table S3.** The relative transmittance of each light source in algal fluid with different densities of algal cells (thickness of algal fluid is 30mm).

| Algal cell density(cells/mL) | 1.70E+06 | 3.00E+06 | 8.00E+06 | 1.70E+07 |
| --- | --- | --- | --- | --- |
| LCPl | 100.00% | 100.00% | 100.00% | 100.00% |
| RCPL | 100.87% | 101.44% | 101.93% | 101.59% |
| m=+32 vortex light | 100.47% | 101.23% | 103.77% | 105.27% |
| m=-32 vortex light | 101.75% | 102.85% | 105.73% | 106.46% |
| RCPL-LCPL | 0.869% | 1.445% | 1.928% | 1.592% |
| RVL-LVL(m=±32) | 1.278% | 1.617% | 1.956% | 1.191% |

In the aforementioned three tables, the term RCPL-LCPL denotes the disparity in relative transmittance between RCPL and LCPL in the Chlorella algae fluid. Similarly, RVL-LVL (M=±32) represents the discrepancy in relative transmittance between Right Vortex Light(RVL) with a topological charge m=-32 and Left Vortex Light(LVL) with a topological charge m=+32 in the Chlorella algae fluid.

Through the analysis of these two rows of data in the table, we have identified certain patterns. For instance, in specific conditions, the transmittance of right-handed light in the algal solution exceeds that of left-handed light. This finding has been utilized to generate Figure 3(a) presented in the main text. Additionally, it was observed that light possessing an Orbital Angular Momentum (OAM) exhibits higher transmittance in the algal solution compared to light having solely a Spin Angular Momentum (SAM). Based on this observation, Figure 3(b) has been constructed in the main text.

**Note S3. Details of Chlorella Culture Experiment in the main text**

The experimental conditions are clearly described in the main text. In this section, we mainly provide two line charts to illustrate the data trends throughout the cultivation process of Chlorella, namely the complete data in Tables 1 and 2 of the main text.


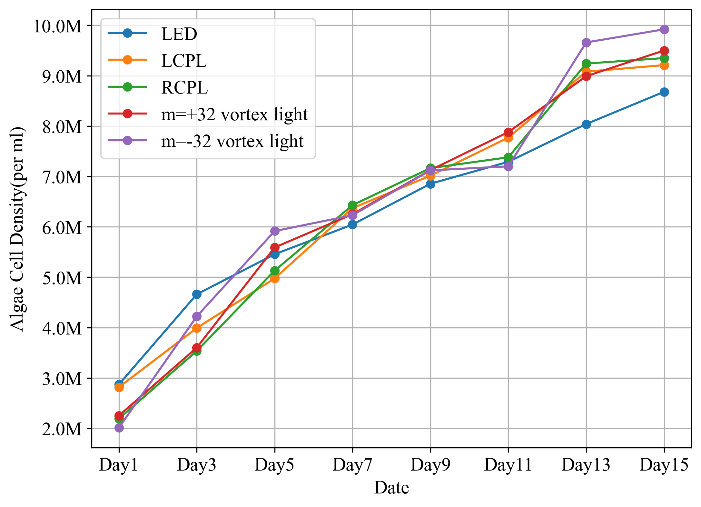


Fig S1. The cell density Line chart of Chlorella


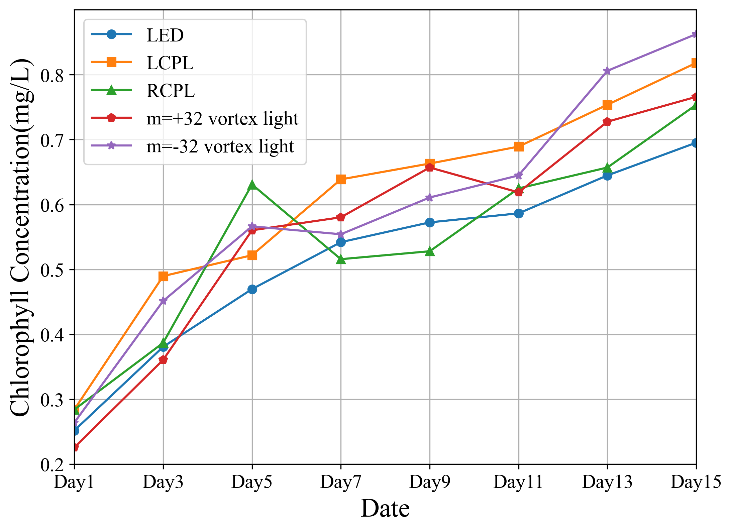


Fig S2. The chlorophyll concentration Line chart of Chlorella

**Note S4. Other supplementary data**

The Excel document linked in this section contains the data utilized for generating the figures presented in the main body of the paper, as well as the complete set of experimental data.

[Data File 1(For Figure3(a)).csv](file:///D:\具有OAM%20的光与蛋白核小球藻藻液相互作用机制的研究\Data%20File%201(For%20Figure3(a)).csv)

[Data File 2(For Figure3(b)).csv](file:///D:\具有OAM%20的光与蛋白核小球藻藻液相互作用机制的研究\Data%20File%202(For%20Figure3(b)).csv)

[Data File 3(For Figure4(b)).csv](file:///D:\具有OAM%20的光与蛋白核小球藻藻液相互作用机制的研究\Data%20File%203(For%20Figure4(b)).csv)

[Data File 4(For Figure4(c)).csv](file:///D:\具有OAM%20的光与蛋白核小球藻藻液相互作用机制的研究\Data%20File%204(For%20Figure4(c)).csv)

[Complete experimental data.xlsx](file:///D:\具有OAM%20的光与蛋白核小球藻藻液相互作用机制的研究\Complete%20experimental%20data.xlsx)

References

1. Kuhn, W. The physical significance of optical rotatory power. *Trans. Faraday Soc.* **26**, 293 (1930).

2. Forbes, K. A. & Jones, G. A. Optical vortex dichroism in chiral particles. *Phys. Rev. A* **103**, 053515 (2021).
